# Rates of paclitaxel hypersensitivity reactions using a modified Markman’s infusion protocol as primary prophylaxis

**DOI:** 10.1007/s00520-024-08460-z

**Published:** 2024-04-17

**Authors:** Rebecca Symons, Fiona Heath, Jennifer Duggan, Kim Tam Bui, Lily Byun, Michael Friedlander, Yeh Chen Lee

**Affiliations:** 1 Royal Hospital for Women, Randwick, NSW Australia; 2 Prince of Wales Hospital, Randwick, NSW Australia; 3 School of Clinical Medicine, Faculty of Medicine and Health, UNSW Sydney, Sydney, NSW Australia

**Keywords:** Paclitaxel hypersensitivity reaction, Gynaecological cancer

## Abstract

**Purpose:**

Markman’s desensitisation protocol allows successful retreatment of patients who have had significant paclitaxel hypersensitivity reactions. We aimed to reduce the risk and severity of paclitaxel hypersensitivity reactions by introducing this protocol as primary prophylaxis.

**Methods:**

We evaluated all patients with a gynaecological malignancy receiving paclitaxel before (December 2018 to September 2019) and after (October 2019 to July 2020) the implementation of a modified Markman’s desensitisation protocol. The pre-implementation group received paclitaxel over a gradually up-titrated rate from 60 to 180 ml/h. The post-implementation group received paclitaxel via 3 fixed-dose infusion bags in the first 2 cycles. Rates and severity of paclitaxel hypersensitivity reactions were compared.

**Results:**

A total of 426 paclitaxel infusions were administered to 78 patients. The median age was 64 years (range 34–81), and the most common diagnosis was ovarian, fallopian tube and primary peritoneal cancer (67%, *n* = 52/78). Paclitaxel hypersensitivity reaction rates were similar in the pre-implementation (8%, *n* = 16/195) and post-implementation groups (9%, *n* = 20/231; *p* = 0.87). Most paclitaxel hypersensitivity reactions occurred within 30 min (pre- vs. post-implementation, 88% [*n* = 14/16] vs. 75% [*n* = 15/20]; *p* = 0.45) and were grade 2 in severity (pre- vs. post-implementation, 81% [*n* = 13/16] vs. 75% [*n* = 15/20]; *p* = 0.37). There was one grade 3 paclitaxel hypersensitivity reaction in the pre-implementation group. All patients were successfully rechallenged in the post-implementation group compared to 81% (*n* = 13/16) in the pre-implementation group (*p* = 0.43).

**Conclusion:**

The modified Markman’s desensitisation protocol as primary prophylaxis did not reduce the rate or severity of paclitaxel hypersensitivity reactions, although all patients could be successfully rechallenged.

## Introduction

Paclitaxel is a commonly used chemotherapeutic agent in many cancers, including gynaecological, breast, lung and gastrointestinal cancers [1]. Paclitaxel hypersensitivity reactions occur in 30–40% of patients without pre-medications [2, 3]. Pre-medication with glucocorticoids, H1-antagonists and H2-antagonists reduces this rate to 5–10% [4, 5]. Paclitaxel hypersensitivity reactions most commonly occur within the first 10–15 min of the infusion, and 95% of reactions occur within the first 2 doses of paclitaxel [3].

The mechanism of paclitaxel hypersensitivity reactions is not entirely understood but is believed to be caused by the solvent Cremophor EL in paclitaxel, which triggers non-IgE-mediated mast cell degranulation [4–7]. A retrospective review explored potential risk factors for paclitaxel hypersensitivity reactions. They identified three factors that were predictive for reactions, which included younger age, history of allergy and short course pre-medication (defined as H1 antagonist, H2 antagonist and dexamethasone 30 min prior to treatment only) [6]. Another review found that the rates of reactions were higher in female patients and in patients with gynaecological malignancies [8].

Markman et al. assessed a three-bag graduated paclitaxel desensitisation protocol in patients who had a clinically significant paclitaxel hypersensitivity reaction and who could not be rechallenged using usual protocols (all patients attempted paclitaxel rechallenge 30 min after the initial paclitaxel hypersensitivity reaction except for one patient due to the severity of the reaction and their underlying comorbidities) [9]. The desensitisation protocol included 9 patients treated between January 1995 and December 1998. All 9 patients successfully received the desensitisation protocol and were able to complete their course of paclitaxel without further hypersensitivity reactions. This protocol also included higher doses and more frequent administration of pre-medications with 20 mg oral dexamethasone 36 h prior to, 12 h prior to and on the morning of treatment as well as 20 mg intravenous dexamethasone, diphenhydramine 50 mg and famotidine 20 mg 30 min prior to treatment. Paclitaxel was infused via three separate infusion bags, containing 2 mg in 100 ml of 0.9% sodium chloride over 30 min, 10 mg in 0.9% sodium chloride over 30 min and then the remainder of the paclitaxel dose in 500 ml of 0.9% sodium chloride over 3 h, respectively. Each subsequent bag was infused if the patient did not react to the preceding infusion of paclitaxel. If the patient experienced a hypersensitivity reaction, the paclitaxel infusion was stopped and rescue medications (intravenous diphenhydramine (50 mg) and hydrocortisone (100 mg)) were administered immediately. The paclitaxel infusion could be reinitiated in approximately 30 min, after the symptoms had subsided. Patients who experienced another reaction at this point would have no further attempt at treatment with paclitaxel. All patients successfully underwent the desensitisation protocol and were able to complete their course of paclitaxel without further incident. This Markman protocol formed the basis of the protocol that we used.

## Methods

We performed an audit of all patients with a gynaecological malignancy who received paclitaxel before (December 2018 to September 2019) and after (October 2019 to July 2020) the implementation of a modified Markman’s protocol, at the Royal Hospital for Women in Randwick, Australia.

### Patient selection

All consecutive patients who received chemotherapy at the chemotherapy day unit over the period of December 2018 to July 2020 were identified via electronic records. Patients were included if they were over the age of 18 years, had a histologically confirmed diagnosis of a gynaecological cancer regardless of cancer stage and were receiving paclitaxel (either 60–80 mg/m^2^ weekly or 175 mg/m^2^ every 21 days) either as a single agent or in combination with other systemic therapy.

### Data collection

Data was collected by reviewing the electronic medical records, which included clinical notes and pharmacy records. The following information was extracted: patient age, cancer details (including type of cancer and stage of cancer), treatment details (including chemotherapy regimen, line of treatment and pre-medications), infusion details (including duration of infusion and cost of paclitaxel infusion) and paclitaxel hypersensitivity reaction details (including severity of reaction and rescue medications given during a reaction).

### Paclitaxel regimens

Patients received paclitaxel as part of a weekly or 21-day regimen at the discretion of their treating oncologist. Patients receiving weekly paclitaxel started with a dose of 60–80 mg/m^2^, and patients receiving paclitaxel every 21 days started at 175 mg/m^2^. For patients in both the pre-implementation and post-implementation groups who had a significant paclitaxel hypersensitivity reaction and were changed to docetaxel, only the doses of paclitaxel were included in the analysis.

### Pre-implementation group

Patients in the pre-implementation group received paclitaxel as a single bag infusion gradually up-titrated from a rate of 60 to 180 ml/h over a duration of 1–3 h depending on what dose of paclitaxel was being administered. Pre-medication, monitoring and management of paclitaxel hypersensitivity reactions were part of the standard procedure in the chemotherapy unit [10]. All patients received pre-medications including famotidine 40 mg and loratadine 10 mg at least 60 min prior to administration of paclitaxel as well as oral dexamethasone 8 mg the evening prior to and the morning prior to chemotherapy. These pre-medications remained the same for patients receiving paclitaxel every 21 days or who had previously had a paclitaxel hypersensitivity reaction. The pre-medications were gradually weaned and ceased after 3 weeks in patients receiving weekly paclitaxel infusions who had not had a paclitaxel hypersensitivity reaction. All patients received education on symptoms of hypersensitivity, and their vital sign observations were recorded prior to paclitaxel infusion. Patients were monitored for symptoms of hypersensitivity throughout the paclitaxel infusion period in the chemotherapy day unit.

Patients who developed a paclitaxel hypersensitivity reaction had their infusion ceased immediately and received hydrocortisone and/or promethazine depending on the severity of the reaction (graded using the Common Terminology Criteria for Adverse Events (CTCAE) v5.0) [11]. Patients who had a paclitaxel hypersensitivity reaction were rechallenged following physician assessment if the reaction was grade 2 or less in severity, and the symptoms had resolved after 30 min.

### Post-implementation group

The published Markman’s protocol was modified to retain the same pre-medications for the pre-implementation group given the protocol was being used as primary prophylaxis (Table 1). Paclitaxel infusions were delivered using the three-bag infusion regimen of Markman’s desensitisation protocol for the first two paclitaxel infusions. The first bag contained 2 mg of paclitaxel in 100 ml of 0.9% sodium chloride and was administered over 30 min. If there was no reaction to the first bag, the second bag containing 10 mg of paclitaxel in 100 ml of 0.9% sodium chloride was administered over 30 min. If there was no reaction to the second bag, the third and final bag containing the remainder of the paclitaxel dose was administered over 1–3 h depending on the dose [9]. Patients who had a paclitaxel hypersensitivity reaction were treated and rechallenged the same as for the pre-implementation group.

**Table 1:** Three-bag infusion protocol based on Markman’s desensitisation protocol

Bag 1	Bag 2	Bag 3
Paclitaxel 2 mg in 100 ml of saline over 30 min	Paclitaxel 10 mg in 100 ml of saline over 30 min	Remainder of paclitaxel dose in 500 ml of saline over 1-3 h (depending on the dose)

### Data analysis

Statistical analysis was performed using SPSS version 23. Descriptive analysis was performed using means, medians and ranges to compare age, diagnosis, treatment regimen, line of therapy and duration of infusion. For analysis of rates and severity of paclitaxel hypersensitivity reactions, a chi-square test was performed, with a *p* value < 0.05 used to signify statistical significance. Cost was obtained from pharmacy invoice records and directly compared between the two groups.

## Results

Between December 2018 and September 2019, a total of 195 doses of paclitaxel were administered to 40 patients and were analysed as part of the pre-implementation group. The modified Markman desensitisation protocol was implemented and used in all patients receiving a paclitaxel infusion after 1 October 2019. From October 2019 to July 2020, a total of 231 doses of paclitaxel were administered to 38 patients and were analysed as part of the post-implementation group.

The baseline characteristics were similar between the two groups (Table 2). The overall median age was 64 years. The most common diagnosis in both groups was ovarian, fallopian tube and primary peritoneal cancer, followed by endometrial cancer. Fifty percent of patients in the pre-implementation group and 58% of patients in the post-implementation group were receiving the first or second dose of paclitaxel as part of their first line of therapy. Most patients received a platinum doublet regimen with carboplatin and paclitaxel. Other regimens included paclitaxel in combination with carboplatin and bevacizumab, with tremelimumab and durvalumab as part of a clinical trial and with carboplatin and trastuzumab. Most patients received paclitaxel given once every 21 days. One patient in the pre-implementation group and one patient in the post-implementation group changed to docetaxel following one dose of paclitaxel due to paclitaxel hypersensitivity reactions.

**Table 2:** Baseline characteristics of patients receiving paclitaxel chemotherapy pre- and post-implementation of a modified Markman’s desensitisation protocol as prophylaxis

	Pre-implementation (*N* = 40)	Post-implementation (*N* = 38)
Age (median (range)) years	66 (37–79)	62.5 (34–81)
Diagnosis		
Ovarian, fallopian tube and primary peritoneal cancer	26	26
Endometrial carcinoma	9	9
Cervical carcinoma	2	1
Cancer of gynaecological origin, not otherwise specified	2	1
Vaginal carcinoma	1	1
Regimen		
3-weekly paclitaxel	30	22
Weekly paclitaxel*	10	16
Line of therapy		
First	31	26
Second	3	4
Third or later	6	8

^*^1 patient in the pre-implementation and 1 patient in the post-implementation groups were initially treated with carboplatin AUC2 in combination with paclitaxel 60 mg/m^2^ and then changed to carboplatin AUC5 in combination with paclitaxel 175 mg/m^2^ from cycle 2. The 21-day paclitaxel regimen dose started at 175 mg/m^2^ and the weekly paclitaxel dose started at 60 mg/m^2^ or 80 mg/m^2^

### Paclitaxel hypersensitivity reaction rate, severity and characteristics

In the pre-implementation group, 16 episodes of paclitaxel hypersensitivity reactions occurred in 11 patients (Table 3). Five of the 11 patients experienced multiple reactions. Most reactions occurred in the first (*n* = 3) and second (*n* = 7) infusions with 6 reactions occurring in the subsequent infusions. Of the paclitaxel hypersensitivity reactions occurring beyond the 2nd infusion, 2 patients had prior reactions in the first or second infusion and 3 patients did not have a recorded reaction to a prior infusion. In the post-implementation group, 20 episodes of paclitaxel hypersensitivity reactions occurred in 12 patients (Table 3). Six of the 12 patients experienced multiple reactions. Again, most reactions occurred during the first (*n* = 4) and second (*n* = 7) infusions with 9 reactions occurring in subsequent infusions. Of the patients who experienced a paclitaxel hypersensitivity reaction after the 2nd cycle, 6 had previously had a reaction during their first or second infusion and 3 did not have a recorded reaction to a prior infusion. Paclitaxel hypersensitivity reaction rates were similar in the pre-implementation (8%, *n* = 16/195) and post-implementation groups (9%, *n* = 20/231; *p* = 0.87) (Table 3). When comparing the rates of reactions during the first or second infusions only, there was no significant difference between the two groups (pre-implementation group 20% (*n* = 10/49) vs. post-implementation group 20% (*n* = 11/53); *p* = 0.97).

**Table 3:** Number and rates of paclitaxel hypersensitivity reactions

	Pre-implementation infusions (*N* = 195)	Post-implementation infusion (*N* = 231)	*P* value
Rate of paclitaxel hypersensitivity reaction *N* (%)	16 (8)	20 (9)	0.87

**Fig. 1:**
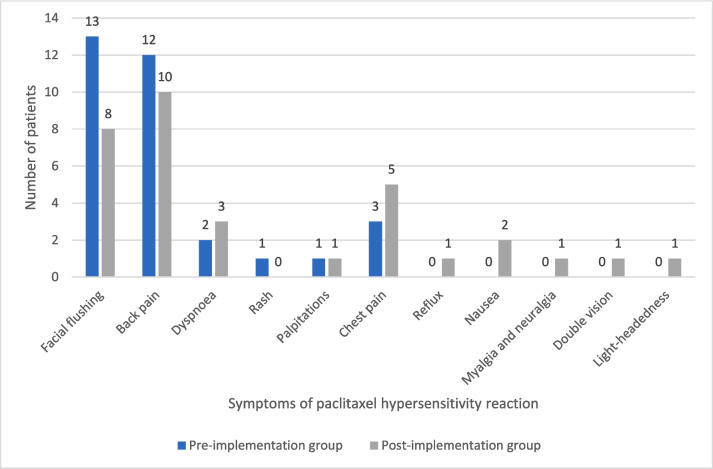
Symptoms of paclitaxel hypersensitivity reactions, pre- and post-implementation of modified Markman’s infusion protocol as a prophylaxis measure

Most paclitaxel hypersensitivity reactions occurred within 30 min of the infusion, when the rate was 60 ml/h in the pre-implementation group (88%; *n* = 14/16) and during the first bag containing 2 mg of paclitaxel in the post-implementation group (75%; *n* = 15/20) (*p* = 0.45). No patients had a delayed reaction in the pre-implementation group. One patient in the post-implementation group developed a rash 1 week after the infusion which was attributed to paclitaxel. As shown in Table 4, most reactions in both groups were grade 2 in severity (pre- vs. post-implementation, 81% [*n* = 13/16] vs. 75% [*n* = 15/20]; *p* = 0.37) (graded using CTCAEv5.0) [11]. One patient in the pre-implementation group had a grade 3 reaction. There were no grade 3 reactions in the post-implementation group and no grade 4 reactions in either group. Rescue medication with IV hydrocortisone 100 mg and/or IV promethazine 12.5 mg was required in 81% of the reactions in the pre-implementation group (*n* = 13/16) and 70% of the reactions in the post-implementation group (*n* = 14/20). All patients were successfully rechallenged in the post-implementation group compared to 81% (*n* = 13/16) in the pre-implementation group (*p* = 0.43). The reasons for not rechallenging the 3 patients in the pre-implementation group were due to a grade 3 reaction in one patient and physician choice for the other 2 patients. The symptoms reported during a reaction are outlined in Fig. 1 and include facial flushing, back pain, chest pain, dyspnoea, palpitations and rash.

**Table 4:** Severity of paclitaxel hypersensitivity reactions

Severity (CTCAE v5.0)	Pre-implementation hypersensitivity reactions (*N* = 16)	Post-implementation hypersensitivity reactions (*N* = 20)
Grade 1	2	5
Grade 2	13	15
Grade 3	1	0
Grade 4	0	0

### Duration and cost

The mean duration of the paclitaxel infusion was similar between the two groups (pre-implementation group 2 h 17 min (range 1 h 4 min–5 h 52 min); post-implementation group (2 h 16 min (range 51 min–6 h 18 min)).

The cost of the paclitaxel infusion in the pre-implementation group depended on the dose. The cost of the infusion in the post-implementation group was also dependent on the dose but was approximately AUD$100 more per dose for the first two paclitaxel infusions due to the split bag regimen, compared to the pre-implementation group. Bag 1 (containing 2 mg of paclitaxel in 100 ml of 0.9% sodium chloride) cost AUD$54.11, bag 2 (containing 10 mg of paclitaxel in 100 ml of 0.9% sodium chloride) cost AUD$55.43 and bag 3 was almost identical to the cost of the single bag in the pre-implementation group for the same dose.

## Discussion

The rates and severity of paclitaxel hypersensitivity reactions remained similar despite the use of a modified Markman’s protocol as primary prophylaxis compared to a standard 3-h infusion regimen. The rate of paclitaxel hypersensitivity reactions in our study was consistent with the literature, with reported rates ranging from 5 to 15% with the use of pre-medications [1, 3, 6, 8, 9, 12–17]. Although the incidence of hypersensitivity reactions was similar with modified Markman’s protocol to our standard 3-h infusion protocol, there were no grade 3 hypersensitivity reactions, and all patients who experienced reactions were able to be successfully rechallenged using a modified Markman’s protocol. Nonetheless, given the additional cost and similar rate of hypersensitivity reactions, Markman’s protocol should be reserved as a rechallenge strategy for patients who have previously had a paclitaxel hypersensitivity reaction, using the higher doses of dexamethasone and antihistamines originally reported.

Paclitaxel is a key chemotherapy agent used in the treatment of many cancers, especially gynaecological cancers. Therefore, any effort toward minimising the rates of paclitaxel hypersensitivity reactions has major clinical implications for the treatment of these patients. Pre-medications with dexamethasone and H1 and H2 antagonists have been highly successful in preventing severe or life-threatening paclitaxel hypersensitivity reactions [1]. However, there is much variation in clinical practice regarding the use of paclitaxel pre-medications. In our study, the same pre-medications were used in the pre-implementation and post-implementation groups. Multiple studies have analysed different pre-medications in both weekly paclitaxel and paclitaxel every 21 days with mixed results [6, 12–16, 19]. A meta-analysis which included 28 studies assessing the use of pre-medications in both weekly and 21-day paclitaxel regimens found that a tapering dexamethasone regimen in patients without hypersensitivity reactions after the first weekly dose of paclitaxel is safe. It also found that a single dose of 20 mg IV dexamethasone instead of the standard oral 20 mg dexamethasone regimen prior to the administration of 21-day paclitaxel is likely to be associated with a higher rate of hypersensitivity reactions, suggesting that multiple doses of dexamethasone pre-medication may have a role in reducing the rate of paclitaxel hypersensitivity reactions [12]. A double-blind randomised controlled trial compared the efficacy and side effects of intravenous 20 mg versus oral 20 mg dexamethasone pre-medication for 281 patients receiving paclitaxel for gynaecological cancers, with no significant difference in rates of reactions [18]. Another retrospective study included women receiving paclitaxel 175 mg/m^2^, 93 of whom received oral 20 mg dexamethasone and 55 of whom received IV 20 mg dexamethasone [13]. The rate of hypersensitivity reactions was 5.4% in the oral dexamethasone group compared to 14.5% in the IV dexamethasone group.

To our knowledge, no studies have utilised Markman’s desensitisation protocol as a primary prophylaxis measure with the aim of reducing the rate of paclitaxel hypersensitivity reactions. However, a prospective study of 222 first- and second-lifetime exposure to paclitaxel and docetaxel infusions comparing a three-step titration method compared to a non-titration method did show a significant reduction in hypersensitivity reactions (19% in the non-titrated group compared to 7% in the titrated group) [20]. This study was also a small (*n* = 222 infusions) single-centre study that used a titrated infusion method, which differed from the three-bag titration method used in our study. While our study did not show a significant difference in the rate of paclitaxel hypersensitivity reactions, this study did. Further studies assessing titration methods with the aim of reducing the rate of paclitaxel hypersensitivity reactions are required. Multiple other studies have been conducted to assess the effectiveness of other interventions in reducing the rate of paclitaxel hypersensitivity reactions, with varying results. A study that assessed the effectiveness of a test dose program with taxanes on hypersensitivity reactions and cost included 206 patients receiving either paclitaxel or docetaxel from 1998 to 2000 [4]. They found that the rate of hypersensitivity reactions was comparable between the two groups. Another study also assessed the cost-effectiveness of a test dose program for paclitaxel to reduce drug wastage related to infusion reactions and included 162 patients who received paclitaxel prior to the implementation of the test dose, from January 1997 until February 2003, 10 of whom developed a hypersensitivity reaction [16]. The test dose was then implemented (a single 12 mg dose of paclitaxel given at a rate of 2 mg/min), and 130 patients who received 244 test doses from June 2003 to March 2005 were included in the study. They found a 63% reduction in paclitaxel hypersensitivity reactions but a 29% increase in the cost. A study assessing the impact of infusion time on hypersensitivity reactions compared two cohorts of patients: one group that received a titrated dose of paclitaxel (*N* = 143) and one group that did not (*N* = 46) [21]. They found that a slow or titrated infusion rate did not mitigate hypersensitivity reactions and that it was associated with an increased likelihood of infusion reactions during the first two dose administrations. A limitation was that there were relatively few patients in the standard rate cohort. A retrospective, single-centre review compared the use of rescue medications in two cohorts of patients: one receiving infusion rate escalation (*N* = 77) and the other cohort receiving a standard infusion (*N* = 22) rate [22]. The use of rescue medications was 23% in the rate escalation infusion cohort and 5% in the standard infusion cohort [22].

For patients who have had a previous paclitaxel hypersensitivity reaction, paclitaxel desensitisation is an option and commonly used except in patients who experienced a severe life-threatening immunocytotoxic reaction such as Stevens-Johnson syndrome, toxic epidermal necrolysis or drug-induced eosinophilia and systemic symptoms (DRESS) [3]. Skin testing has also been studied but is not validated for taxanes as the mechanism of hypersensitivity reactions is not thought to be primarily IgE mediated [17]. In patients who have had a significant hypersensitivity reaction to paclitaxel, there are alternative options such as docetaxel, although some studies have reported a cross-reactivity rate of up to 90% [1, 5]. Another alternative is the use of nanoparticle albumin-bound paclitaxel (nab-paclitaxel) as the formulation with albumin allows reconstitution of nab-paclitaxel with a saline solution instead of solvents, has lower rates of hypersensitivity and therefore does not require pre-medication with corticosteroids, although there are no studies showing that nab-paclitaxel is safe in patients who have previously had a grade 3 or 4 paclitaxel hypersensitivity reaction [23].

In our audit, for the data collected retrospectively from the pre-implementation period, there was unavoidably missing data for infusion time and assessment of paclitaxel hypersensitivity reactions. However, the variations in assessment appeared to be minimal and did not affect the quality of the data collected overall. The implementation of modified Markman’s infusion was then protocolized, and the data for the post-implementation group was prospectively collected to ensure a quality assessment of hypersensitivity reactions during this period. Since our analysis only focused on the female population with gynaecological cancers, the results may not be generalisable to the male population and other types of cancers. It is also important to acknowledge the inherent limitations of conducting a single-centre retrospective study. Firstly, although our results provide valuable insights into the outcomes within our centre, caution should be exercised when extrapolating these findings to different settings or populations. The unique characteristics of our patient population and the specific treatment protocols utilised here may not be directly applicable to other contexts. Secondly, we acknowledge that the sample size of our study may limit the ability to detect small effect sizes. This limitation underscores the need for cautious interpretation of our findings and highlights the need for additional studies to validate and expand upon our results.

We did not observe a significant difference in the rate of paclitaxel hypersensitivity reactions using a modified Marman’s desensitisation protocol as a primary prophylaxis measure. The management and prevention of hypersensitivity reactions remain an important issue in the management of multiple malignancies, particularly gynaecological malignancies where paclitaxel is frequently used. Furthermore, studies on variations to pre-medications have not consistently yielded an improvement in the rates of paclitaxel hypersensitivity reactions. Given the time and cost for the administration, Markman’s desensitisation protocol with higher dose steroids should be reserved for those with paclitaxel hypersensitivity reactions who are not successfully rechallenged and for whom paclitaxel is considered essential.

## References

[1] Zidan J, Hussein O, Abzah A, Tamam S, Farraj Z, Friedman E (2008). Oral premedication for the prevention of hypersensitivity reactions to paclitaxel. Med Oncol.

[2] Berger MJ, Dulles LJ, Rettig AE, Lustberg MB, Phillips GS, Shapiro CL (2012). Feasibility of stopping paclitaxel premedication after two doses in patients not experiencing a previous infusion reaction. Support Care Cancer.

[3] Boulanger J, Boursiquot JN, Cournoyer G, Lemieux J, Masse MS, Almanric K, the Comite de l’evolution des pratiquesenoncologie (2014). Management of hypersensitivity to platinum- and taxane-based chemotherapy: CEPO review and clinical recommendations. Curr Oncol.

[4] Caiado J, Castells MC (2021). Drug desensitizations for chemotherapy: safety and efficacy in preventing anaphylaxis. Curr Allergy Asthma Rep.

[5] Mezzano V, Giavina-Bianchi P, Picard M, Caiado J, Castells M (2014). Drug desensitization in the management of hypersensitivity reactions to monoclonal antibodies and chemotherapy. BioDrugs.

[6] Aoyama T, Takano M, Miyamoto M, Yoshikawa T, Soyama H, Kato K, Ishibashi H, Iwahashi H, Nakatsuka M, Yajima I, Shimizu Y (2017). Is there any predictor for hypersensitivity reactions in gynecologic cancer patients treated with paclitaxel-based therapy?. Cancer Chemother Pharmacol.

[7] Rodríguez-Antona C (2010). Pharmacogenomics of paclitaxel. Pharmacogenomics.

[8] Lansinger OM, Biedermann S, He Z, Colevas AD (2021). Do steroids matter? A retrospective review of premedication for taxane chemotherapy and hypersensitivity reactions. J Clin Oncol.

[9] Markman M, Kennedy A, Webster K, Kulp B, Peterson G, Belinson J (2000). Paclitaxel-associated hypersensitivity reactions: experience of the gynecologic oncology program of the Cleveland Clinic Cancer Center. J Clin Oncol.

[10] EviQ protocol ID 3264 v.3: Premedication for prophylaxis of taxane hypersensitivity reactions (infusion related reactions and anaphylaxis). https://www.eviq.org.au/clinical-resources/side-effect-and-toxicity-management/immunological/3264-premedication-for-prophylaxis-of-taxane-hyper. Accessed 5^th^ Feb 2024

[11] National Cancer Institute (2017) Common Terminology Criteria for Adverse Events (CTCAE) version 5.0. National Cancer Institue. https://ctep.cancer.gov/protocoldevelopment/electronic_applications/ctc.htm. Accessed: 24th April 2023

[12] Dewaele E, Verschueren C, Specenier P (2017). Premedication strategy for paclitaxel, still an unsolved question after 30 years. Belg J Med Oncol-Amsterdam, 2007, Currens.

[13] O’Cathail SM, Shaboodien R, Mahmoud S, Carty K, O’Sullivan P, Blagden S, Kwon JS, Agarwal R (2013). Intravenous versus oral dexamethasone premedication in preventing Paclitaxel infusion hypersensitivity reactions in gynecological malignancies. Int J Gynecolo Cancer.

[14] Yenilmez A, Hood AP, Nguyen LH, Merl MY (2017). Paclitaxel pre-medication: a comparison of two steroid pre-medication protocols. J Oncol Pharm Pract.

[15] Krieger JA, Stanford BL, Ballard EE, Rabinowitz I (2002). Implementation and results of a test dose program with taxanes. J Cancer.

[16] Henry A, Charpiat B, Perol M, Vial T, de Saint Hilaire PJ, Descotes J (2006). Paclitaxel hypersensitivity reactions: assessment of the utility of a test-dose program. J Cancer.

[17] Tsao LR, Young FD, Otani IM, Castells MC (2021). Hypersensitivity reactions to platinum agents and taxanes. Clin Rev Allergy Immunol.

[18] Yanaranop M, Chaithongwongwatthana S (2016). Intravenous versus oral dexamethasone for prophylaxis of paclitaxel-associated hypersensitivity reaction in patients with primary ovarian, fallopian tube and peritoneal cancer: a double-blind randomized controlled trial. Asia-Pac J Clin Oncol.

[19] Noronha V, Enting D, Thippeswamy R, Joshi A, Patil VM, Prabhash K (2018). Hypersensitivity reactions to paclitaxel with a modified dexamethasone intravenous premedication regimen. Cancer Res Stat Treat.

[20] Lynch DM, Menon S, Mazzola E, Costa J, Jabaley T (2023). A three-step taxane titration protocol decreases hypersensitivity reactions during first and second exposures. JCO Oncol Pract.

[21] Ferrigni E, Grate L, Herzog T, Billingsley C, Jackson A (2021). Comparison of incidence of hypersensitivity reactions of paclitaxel infusions with titrated versus non-titrated infusions. Gynecol Oncol.

[22] Nymberg K, Folan S, Berger M, Li J, Zanath K, VanDeusen J, Vargo C (2021). Comparison of subsequent infusion hypersensitivity reactions to paclitaxel using two different infusion strategies. Support Care Cancer.

[23] Gradishar WJ (2006). Albumin-bound paclitaxel: a next-generation taxane. Expert Opin Pharmacother.

